# Evaluation of Immunomodulatory and Antiarthritic Potential of *Trigonella gharuensis* Extracts

**DOI:** 10.1155/2020/8836080

**Published:** 2020-12-12

**Authors:** Aisha Mobashar, Arham Shabbir, Muhammad Shahzad, Saeed-ul-Hassan

**Affiliations:** ^1^Faculty of Pharmacy, The University of Lahore, Lahore, Pakistan; ^2^Institute of Pharmacy, Faculty of Pharmaceutical and Allied Health Sciences, Lahore College for Women University, Jail Road, Lahore, Pakistan; ^3^Department of Pharmacology, University of Health Sciences, Lahore, Punjab, Pakistan; ^4^Imran Idrees College of Pharmacy, 3 km Daska Road, Sialkot, Pakistan

## Abstract

The genus of *Trigonella* has long been used for the treatment of arthritis and inflammatory disorders. This study was aimed to investigate the immunomodulatory activities of ethanol and *n*-hexane extracts of *T. gharuensis* in the rat model of rheumatoid arthritis. Freund's complete adjuvant (FCA) model was used to induce arthritis in rats. Arthritis was induced on day 0, while treatment which was started on day 8 continued for twenty days. Arthritic development and paw edema were determined using an arthritic scoring index and plethysmometer, respectively. Histopathology was evaluated using H&E staining. RNA extraction, reverse transcription, and polymerase chain reaction (RT-PCR) were performed to determine expression levels of proinflammatory markers such as TNF-*α*, NF-*ĸ*B, IL-6, IL-1*β*, COX2, and anti-inflammatory cytokine IL-4. Prostaglandin E2 level (PGE2) was evaluated using ELISA. Blood analysis and biochemical parameters were also determined. The significance level was set as *P* < 0.05. Treatment with extracts reduced paw edema, arthritic progression, and histopathological parameters. Expression levels of abovementioned proinflammatory cytokines and COX2 were downregulated, while IL-4 was upregulated. PGE2 levels were found reduced with extract treatment. Blood parameters were nearly normalized in treatment groups. Extract treatment did not alter biochemical parameters. Both extracts had effects comparable with piroxicam. In conclusion, extracts of *T. gharuensis* ameliorated experimentally induced arthritis that may be ascribed to its immunomodulatory effects.

## 1. Introduction

Rheumatoid arthritis (RA) is a chronic inflammatory and autoimmune disorder that presents with bone erosion, cartilage damage, and inflammation. Major symptoms include varying degrees of joint stiffness, pain, and swelling, while systemic symptoms include fever, fatigue, and weight loss [1].

Various cytokines and proinflammatory mediators are involved in the pathogenesis of RA. These cytokines are released by activated macrophages and fibroblasts. Tumor necrosis factor (TNF-*α*) stimulates IL-6 which is responsible for activation of chondrocytes and fibroblasts in articular cartilage. This leads to erosion of collagen and proteoglycans resulting in joint destruction. IL-1*β* in diseased synovial membrane is considered responsible for pannus formation and bone erosion. IL-6 stimulates production of nuclear factor kappaB (NF-*ĸ*B) which, in turn, activates osteoclasts, abnormal apoptosis, and proliferation of synovial cells [2]. Likewise, augmented levels of COX2 activate PGE2 production which leads to angiogenesis and articular cartilage destruction along with pain and inflammation [3].

Nonsteroidal anti-inflammatory drugs (NSAID) and opiates have been extensively used for the symptomatic treatment of arthritis [4]. Adverse effects of NSAIDs include gastric perforation and bleeding due to reduced prostaglandin production [5]. Continued use of opiates poses a serious risk of respiratory depression, dependence, and tolerance [6]. Corticosteroids are associated with peptic ulcer, osteoporosis, precipitation of diabetes, and higher susceptibility to infections due to immunosuppressive properties [7]. Other drugs used to treat autoimmune disorders, such as DMARDs and cytokines antagonist, have been associated with increased susceptibility to infections seen in the patients [8].

Due to many adverse effects of synthetic drugs, modern research focuses on the use of medicinal plants for treatment of inflammation and arthritis due to their easy availability and lower cost value [9]. *Trigonella gharuensis* is an herb of worldwide distribution [10]. It belongs to the second largest family of flowering plants, i.e., fabeaceae [11]. Our previous study has established anti-inflammatory properties of *T. gharuensis* using different paw edema models of acute inflammation such as carrageenan, histamine, serotonin-induced paw edema models, and xylene-induced ear edema model. Moreover, GCMS analysis in our study showed that both extracts possessed considerable constituents responsible for anti-inflammatory and antioxidant properties [12]. Our current study focuses on antiarthritic potential of *T. gharuensis* using FCA-induced chronic model of inflammation in arthritic rats.

## 2. Methodology

### 2.1. Plant Collection and Identification

*T. gharuensis* was collected from Quetta district of Balochistan. It was identified by Department of Botany, University of Balochistan (UOB), Quetta, Pakistan. The voucher specimen (TG-RBT-05) was kept in the herbarium of university [12].

### 2.2. Preparation of Extracts

Herb was dried under shade. It was then chopped followed by grinding. Maceration was done by soaking powder into ethanol and *n*-hexane, respectively. The mixtures were kept at 25°C. The mixtures were filtered and then concentrated at 37°C in a rotary evaporator (IKA Germany) under reduced pressure. After that, extracts were dried in an incubator at 40°C. The EETG (ethanolic extract of *T. gharuensis*) and NHTG (*n*-hexane extract of *T. gharuensis*) were dissolved in 1% Tween 80 before administration.

### 2.3. Test Animals

Sprague Dawley rats (6–8 weeks old) were placed in animal house of The University of Lahore, Lahore. They were acclimatized with environment for one week under controlled humidity conditions (60–70%) and temperature 25°C ± 2, respectively. Food and water access was freely provided. Light and dark cycles were maintained. Approval from the Institutional Research Ethics Committee, The University of Lahore (IREC-2017-23), was taken for the experiment.

### 2.4. Evaluation of Antiarthritic Activities

Thirty rats were distributed into 5 groups. Vehicle control (group 1) and arthritic control (group 2) were given vehicle, i.e., 1% Tween 80 in water [13]. EETG (group 3) and NHTG (group 4) were given extracts (400 mg/kg b.w., p.o., each) [14]. Piroxicam was given (10 mg/kg b.w., i.p.,) to group 5. FCA (0.15 ml) was injected into left paws of all the animals (subplantar region) except vehicle control group at 0 day. Animals were treated with extracts, and piroxicam was started from day 8 till day 28. Treatment with EETG, NHTG, and piroxicam was commenced at day 8 and ended on day 28 [15].

### 2.5. Arthritic Score Measurement

Different parameters such as inflammation, redness, and erythema were observed on different day intervals of 8, 15, 22, and 28 using macroscopic criteria. Score 0 was given to normal, score 1 to minimal, score 2 to mild, score 3 to moderate, and score 4 to severe changes [16].

### 2.6. Paw Volume Assessment

Digital water displacement plethysmometer was used to measure paw edema of all groups at days 0, 8, 15, 22, and 28.

### 2.7. Histopathological Evaluation

On day 28, all rats were sacrificed. Ankle joints were cut longitudinally and placed in formalin (10%) for fixation. Formic acid was used for decalcification of samples. Tissues were cut into thin slices, and paraffin wax was used for embedding the tissues. H&E (hematoxylin and eosin) staining was used. The slides were examined by a blinded histopathologist for presence of pannus formation, bone erosion, and inflammation. Scoring criteria were followed as mentioned previously [15].

### 2.8. Determination of mRNA Expression Levels of TNF-*α*, NF-*κ*B, IL-6, IL-1*β*, COX2, and IL-4

Conventional PCR was used to assess mRNA expression levels of cytokines involved in pathogenesis of RA. First, blood samples were taken for RNA extraction, and it was achieved using TRizol reagent according to protocol. The extracted RNA samples were quantified through a nanodrop spectrophotometer. cDNA was synthesized using kit manufacturer's protocol (Thermo Scientific, America). GAPDH was used as reference. Primers of TNF-*α* and GAPDH were designed manually. Sequence of primers such as IL-6, IL-1*β*_,_ NF-*κ*B, IL-4, and COX2 was selected from previously published studies [17–19], as mentioned in Table 1. cDNA (2 *µ*l) was mixed with forward-reverse primer mix (1 *µ*l), nuclease-free water (3 *µ*l), and PCR Master Mix (6 *µ*l). Thermal cycle was programmed for denaturation (95°C for 10 s), annealing (58°C and 60°C for 20 s), and extension (72°C for 30 s) cycles.

### 2.9. Determination PGE2 Levels

PGE2 levels were measured from serum using ELISA kit (Elab Science E-EL-0034 96T). Optical density was measured using ELISA reader (BioTek, ELx-800) 450 nm length.

### 2.10. Evaluation of Hematological Parameters

Blood samples were collected through intracardiac puncture. Automated hematology analyzer (Sysmex XT-1800i) was used to evaluate hemoglobin content and levels of RBCs, WBCs, and platelets.

### 2.11. Biochemical Parameters

Serum was separated from blood. Different biochemical parameters such as urea, creatinine, aspartate aminotransferase (AST), alanine transaminase (ALT), and alkaline phosphatase (ALP) were analyzed by an automated chemistry analyzer (Humalyzer 3500) by following kit manufacturer's protocols (Analyticon Biotechnologies AG, Germany).

### 2.12. Statistical Analysis

All values were expressed as mean ± SEM. Data were analyzed using Graph Pad Prism (v 6.0). One-way ANOVA followed by Tukey's post hoc test was used for comparison. Significance level was observed as *P* < 0.05.

## 3. Results

### 3.1. *T. gharuensis* Inhibited Arthritic Development

The arthritic progression was induced by using FCA, and it was measured through macroscopic criteria. The increased trend of arthritic development was continued in the diseased control group because no treatment was provided to this group.  Table 2 shows that (*P* < 0.001) arthritic score was reduced at days 15, 22, and 28, respectively, with extract treatment as compared to the positive control group. The piroxicam also significantly reduced (*P* < 0.001) arthritic score at different day intervals which is comparable to EETG and NHTG groups.

### 3.2. *T. gharuensis* Prevented Paw Edema

Remarkable edema was observed in the FCA-induced model, and it was measured through digital water plethysmometer on different day intervals such as 15, 22, and 28. The values of vehicle control group were considered zero because there was no induction of disease in this group.  Table 3 shows increased trend in inflammation, and score was continued until the 28th day of this experimental study in the positive control group. EETG, NHTG, and piroxicam treatment significantly (*P* < 0.001) reduced paw edema when compared with the arthritic control group. The results of extracts treatment were comparable to the piroxicam group.

### 3.3. *T. gharuensis* Significantly Reduced Histopathological Parameters

EETG, NHTG, and piroxicam showed significant inhibition of inflammation, bone erosion, and pannus formation in comparison to the arthritic control group (Table 4). H&E staining picture revealed that aggregates of inflammatory cells presented in the arthritic control group were markedly reduced in piroxicam- and extract-treated groups.

### 3.4. *T. gharuensis* Downregulated Proinflammatory and Upregulated Anti-Inflammatory Cytokines

The collected blood sample on the 28th day was processed for mRNA expression levels.  Table 5 shows that significant levels of proinflammatory markers such as TNF-*α*, IL-6, IL-1*β*, NF-*ĸ*B, and COX2 were increased in the positive control group. The augmented levels of these cytokines were markedly reduced (*P* < 0.001) in EETG and NHTG extract- and piroxicam-treated groups in comparison to the positive control group. Levels of anti-inflammatory cytokines such as IL-4 decreased in the positive control group. IL-4 levels were increased in extract- and piroxicam-treated group in contrast to the positive control group.

### 3.5. *T. gharuensis* Significantly Reduced PGE2 Levels

**Figure 1:**
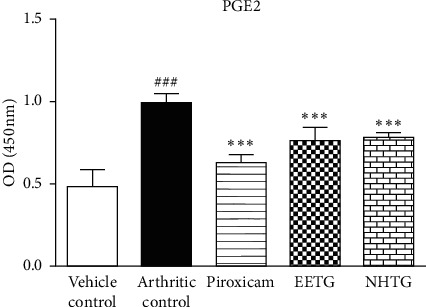
EETG, NHTG (400 mg/kg, each), and piroxicam (10 mg/kg) significantly reduced PGE2 levels as compared to the control group. *∗∗∗P* indicates <0.001 as compared to the arthritic group while ### presents comparison between control and arthritic groups.

Increased (*P* < 0.001) serum PGE2 levels were noticed in the arthritic control group as compared to the vehicle control group. Significant reduction (*P* < 0.001) in PGE2 levels was observed in piroxicam-, EETG-, and NHTG-treated groups, as presented in Figure 1.

### 3.6. *T. gharuensis* Modulated Hematological Parameters

The reduction in hematological parameters, such as RBC and Hb levels, were seen in the positive control group. These parameters were nearly normalized in treatment groups as compared to the positive control group. Similarly, elevated WBC and platelets levels were observed in the arthritic control groups which were nearly normalized after treatment with extracts and the reference drug in contrast to the positive control group (Table 6).

### 3.7. *T. gharuensis* Nearly Normalized Biochemical Parameters

The serum was separated from the blood sample, and different hepatic markers such as ALT  and AST along with renal parameters such as urea and creatinine were measured through commercially available kits.  Table 7 shows that *T. gharuensis* extracts and piroxicam did not alter liver and renal parameters. Statistically nonsignificant differences were found in AST, ALT, urea, and creatinine levels when groups were compared with each other.

## 4. Discussion

Medicinal plants have proved to be safer and cheaper in comparison to synthetic medicines for the treatment of arthritis. Rheumatoid arthritis is characterized by bone damage, deformity, hyperplasia, pannus formation, and inflammation in the joints [20, 21]. The preferred model used for this study is FCA-induced model due to its similarities with arthritic disorder seen in human. Ethanolic and *n*-hexane extracts of *T. gharuensis* extracts are shown to reduce paw edema and arthritic progression. These results are further supported by the histopathological investigations which showed clear amelioration of hallmarks of RA.

After FCA immunization, different cytokines are released by activated macrophages and monocytes which, in turn, release various cytokines such as TNF-*α*, IL-6, and IL-1*β*. These cytokines aggravate inflammation, bone erosion, and cartilage destruction in joint tissues [22]. These cytokines also stimulate NF-*ĸ*B, a transcriptional factor, which promotes bone resorption by activating osteoclasts and proliferation of synovial cells in joints. This factor also worsens the symptoms of RA by favoring Th1 response [17]. Hence, NF-*ĸ*B inhibitors might be useful therapeutically and efficacious in ameliorating symptoms of RA. Our study shows marked decreased in the levels of TNF-*α*, IL-6, NF-*ĸ*B, and IL-1*β* in extract-treated groups in comparison to the arthritic group.

The COX2 and prostaglandins are crucial mediators which are involved in pain and swelling. TNF-*α* and IL-1*β* increase levels of COX2 and PGE2 in activated synovial cells. COX2 also activates process of bone erosion in juxta-articular cartilage. The extracts in current study reduced the levels of COX2 and PGE2 which might be responsible for the amelioration of inflammation and cartilage damage found in the study.

Immunomodulatory potential of *T. gharuensis* is further validated by augmented levels of IL-4 in EETG-, NHTG-, and piroxicam-treated groups in contrast to the arthritic group. One of the reasons for the development of RA is imbalance between the levels of proinflammatory and anti-inflammatory cytokines. Elevation in the levels of proinflammatory cytokines or reduction in the levels of anti-inflammatory cytokines might lead to imbalance and result in development of RA. IL-4 mediates an anti-inflammatory response in RA. IL-4 inhibits Th1 response and favors Th2 immunomodulatory cells. Therapy with recombinant IL-4 has proved to inhibit cytokine production [17, 23]. This study showed significant elevation in the expression levels of IL-4 in extract-treated groups, probably in an attempt to ameliorate Th1 response.

Arthritic patients were found to have altered hematological picture with increased levels of WBCs and platelets, while reduced RBC count and Hb levels. The anemia may be attributed to inadequate erythropoiesis by the bone marrow and disorganized deposition of iron in synovial tissue and reticuloendothelial system [24, 25]. Increased WBC and platelet counts may be ascribed to overactive immune response. Treatment with *T. gharuensis* extracts led to normalisation of these values in the blood picture. In order to determine the safety of the plant extracts, biochemical parameters such as AST, ALT, urea, and creatinine were measured. The increased levels of these markers are indicative of liver injury and kidney dysfunction, respectively. The results showed no significant differences among all the groups which deemed that extracts were safe to use. The inferences of current study are in line with the study of Shabbir et al. [18].

Previously, we published the identification of phytochemical constituents in both plant extracts using GCMS analysis. The data showed the presence of different anti-inflammatory compounds in the extracts, e.g., ethylpalmitate, phytol, *n*-hexadecanoic acid, ethyl linoleate, nanocosane, and coumarin. Presence of these constituents might be responsible for anti-inflammatory activities of both the extracts [12].

## 5. Conclusion

Amelioration of joint inflammation confirmed that *T. gharuensis* extracts possessed significant immunomodulatory and antiarthritic potential in the FCA-induced arthritic rat model. *T. gharuensis* extracts significantly reduced paw edema, arthritic progression, and histopathological parameters. The attenuation of RA might be ascribed to downregulation of proinflammatory markers such as TNF-*α*, IL-1*β*, IL-6, NF-*ĸ*B, and COX2 and upregulation of anti-inflammatory IL-4. Moreover, PGE2 levels were also found reduced after treatment with plant extracts.

## Figures and Tables

**Table 1:** Primer sequences.

Genes	Forward primer	Reverse primer	Product size	Reference
IL-4	5′-CACCTTGCTGTCACCCTGTT-3′	5′-TCACCGAGAACCCCAGACTT-3′	231	[18]
IL-6	5′-CCCACCAAGAACGATAGTCA-3′	5′-CTCCGACTTGTGAAGTGGTA-3′	247	[26]
TNF-*α*	5′-AGTCCGGGCAGGTCTACTTT-3′	5′-GGAAATTCTGAGCCCGGAGT-3′	202	NM_012675.3
NF-*κ*B	5′-TGAGATCCATGCCATTGGCC-3′	5′-AGCTGAGCATGAAGGTGGATG-3′	207	[18]
COX2	5′-CCAGATGGCCAGAGGACTCA-3′	5′-TGTGAGTCCCGAGGGAATAGA-3′	237	[18]
IL-1*β*	5′-GCTGTCCAGATGAGAGCATC-3′	5′-GTCAGACAGCACGAGGCATT-3′	293	[19]
GAPDH	5′-GTCATCAACGGGAAACCCAT-3′	5′-ATCACAAACATGGGGGCATC-3′	197	NM_017008.4

**Table 2:** *T. gharuensis* extracts attenuated arthritic development.

Days	Vehicle control	Arthritic control	Piroxicam	EETG	NHTG
Day 8	0.000 ± 0.000	2.999 ± 0.073	3.003 ± 0.073	3.083 ± 0.083	3.083 ± 0.083
Day 15	0.000 ± 0.000	3.389 ± 0.073	2.245 ± 0.110^*∗∗∗*^	2.833 ± 0.105^*∗∗∗*^	2.833 ± 0.106^*∗∗*^
Day 22	0.000 ± 0.000	3.499 ± 0.122	2.495 ± 0.172^*∗∗∗*^	2.667 ± 0.105^*∗∗*^	2.917 ± 0.153^*∗*^
Day 28	0.000 ± 0.000	3.489 ± 0.149	2.093 ± 0.073^*∗∗∗*^	2.533 ± 0.105^*∗∗∗*^	2.500 ± 0.129^*∗∗∗*^

EETG, ethanolic extract of *T. gharuensis* (400 mg/kg); NHTG, *n*-hexane extract of *T. gharuensis* (400 mg/kg); piroxicam (10 mg/kg). Values were denoted as mean ± SEM. ^*∗*^*P* < 0.05, ^*∗∗*^*P* < 0.01 and ^*∗∗∗*^*P* < 0.001 when compared with the arthritic group.

**Table 3:** *T. gharuensis* extracts significantly reduced paw edema in arthritic rats.

Days	Vehicle control	Arthritic control (ml)	Piroxicam (ml)	EETG (ml)	NHTG (ml)
Day 8	0.000 ± 0.000	0.938 ± 0.012	0.953 ± 0.012	0.937 ± 0.008	0.932 ± 0.014
Day 15	0.000 ± 0.000	1.138 ± 0.015	0.773 ± 0.007^*∗∗∗*^	0.770 ± 0.013^*∗∗∗*^	0.863 ± 0.006^*∗∗∗*^
Day 22	0.000 ± 0.000	1.232 ± 0.010	0.699 ± 0.006^*∗∗∗*^	0.737 ± 0.009^*∗∗∗*^	0.727 ± 0.010^*∗∗∗*^
Day 28	0.000 ± 0.000	1.372 ± 0.008	0.622 ± 0.007^*∗∗∗*^	0.630 ± 0.007^*∗∗∗*^	0.670 ± 0.017^*∗∗∗*^

Value of normal paw is considered as zero. EETG, ethanolic extract of *T. gharuensis*; NHTG, *n*-hexane extract of *T. gharuensis* (400 mg/kg); piroxicam (10 mg/kg). Values were denoted as mean ± SEM. ^*∗∗∗*^*P* < 0.001 when compared with the arthritic control group.

**Table 4:** *T. gharuensis* attenuated histopathological parameters.

Parameters	Vehicle control	Arthritic control	Piroxicam	EETG	NHTG
Infiltration of inflammatory cells	0.000 ± 0.000	2.573 ± 0.073	1.566 ± 0.073^*∗∗∗*^	1.917 ± 0.154^*∗∗∗*^	2.083 ± 0.083^*∗*^
Pannus formation	0.000 ± 0.000	3.405 ± 0.073	2.522 ± 0.103^*∗∗∗*^	2.583 ± 0.083^*∗∗∗*^	2.583 ± 0.083^*∗∗∗*^
Bone erosion	0.000 ± 0.000	2.543 ± 0.073	2.073 ± 0.073^*∗∗*^	2.083 ± 0.083^*∗∗*^	2.167 ± 0.105^*∗*^

Value of normal paw is considered as zero. EETG, ethanolic extract of *T. gharuensis* (400 mg/kg); NHTG, *n*-hexane extract of *T. gharuensis* (400 mg/kg); piroxicam (10 mg/kg). Values were denoted as mean ± SEM. ^*∗∗*^*P* < 0.01 and ^*∗∗∗*^*P* < 0.001 when compared with the arthritic control group.

**Table 5:** *T. gharuensis* downregulated proinflammatory and upregulated anti-inflammatory cytokines.

Markers	Vehicle control	Arthritic control	Piroxicam	EETG	NHTG
TNF-*α*	32.40 ± 1.777	49.97 ± 1.967###	33.72 ± 1.033^*∗∗∗*^	34.29 ± 1.538^*∗∗∗*^	35.38 ± 0.571^*∗∗∗*^
NF-*κ*B	33.01 ± 1.538	52.59 ± 0.679###	34.82 ± 1.226^*∗∗∗*^	39.08 ± 1.365^*∗∗∗*^	41.25 ± 0.690^*∗∗∗*^
IL-6	32.65 ± 1.741	39.61 ± 1.178##	31.99 ± 1.033^*∗∗*^	33.86 ± 0.850^*∗∗*^	33.61 ± 1.195^*∗∗*^
IL-1*β*	33.09 ± 1.462	51.79 ± 1.439###	31.99 ± 1.226^*∗∗∗*^	34.46 ± 1.239^*∗∗∗*^	35.83 ± 0.649^*∗∗∗*^
COX2	33.89 ± 1.699	55.96 ± 1.300###	34.07 ± 0.878^*∗∗∗*^	31.06 ± 0.791^*∗∗∗*^	34.73 ± 0.499^*∗∗∗*^
IL-4	33.95 ± 1.001	22.49 ± 0.505###	29.72 ± 0.724^*∗∗∗*^	31.82 ± 0.551^*∗∗∗*^	33.16 ± 0.427^*∗∗∗*^

EETG, ethanolic extract of *T. gharuensis* (400 mg/kg); NHTG, *n*-hexane extract of *T. gharuensis* (400 mg/kg); piroxicam (10 mg/kg). Values were denoted as mean ± SEM. ^*∗*^*P* < 0.05, ^*∗∗*^*P* < 0.01 and ^*∗∗∗*^*P* < 0.001 when compared with the arthritic control group. ##*P* < 0.01 and ###*P* < 0.001.

**Table 6:** *T. gharuensis* nearly normalized hematological parameters.

Parameters	Control	Arthritic control	Piroxicam	EETG	NHTG
RBC (106/Ul)	8.293 ± 0.310	5.650 ± 0.365###	8.399 ± 0.332^*∗∗*^	7.360 ± 0.365^*∗∗*^	7.337 ± 0.304^*∗∗*^
Hb (g/dl)	13.999 ± 0.129	11.93 ± 0.241###	12.58 ± 0.207^*∗∗*^	14.02 ± 0.425^*∗∗∗*^	13.67 ± 0.353^*∗∗*^
WBC (103/Ul)	10.13 ± 0.325	15.19 ± 0.246###	11.99 ± 0.225^*∗∗∗*^	12.88 ± 0.209^*∗∗∗*^	13.25 ± 0.324^*∗∗*^
Platelets (103/Ul)	782.7 ± 26.82	1434 ± 17.24###	1018 ± 31.31^*∗∗∗*^	910.8 ± 15.84^*∗∗∗*^	1099 ± 34.13^*∗∗∗*^

EETG, ethanolic extract of *T. gharuensis* (400 mg/kg); NHTG, *n*-hexane extract of *T. gharuensis* (400 mg/kg); piroxicam (10 mg/kg). Values were denoted as mean ± SEM. ^*∗∗*^*P* < 0.01 and ^*∗∗∗*^*P* < 0.001 when compared with the arthritic control group. ###Difference between vehicle control group and positive control group.

**Table 7:** Evaluation of biochemical parameters.

Biochemical parameters	Vehicle control	Arthritic control	Piroxicam	EETG	NHTG
Urea (mg/dl)	27.01 ± 0.609	27.10 ± 0.846	25.98 ± 0.391	27.67 ± 0.421	26.17 ± 0.477
Creatinine (mg/dl)	0.829 ± 0.200	0.835 ± 0.014	0.839 ± 0.030	0.866 ± 0.021	0.858 ± 0.020
AST (IU/L)	95.99 ± 1.712	97.88 ± 1.510	93.99 ± 1.182	98 ± 0.617	96.71 ± 1.34
ALT (IU/L)	30.29 ± 0.549	31.63 ± 0.470	33.69 ± 0.321	33.17 ± 0.654	33.17 ± 0.792

Value of normal paw is considered as zero. EETG, ethanolic extract of *T. gharuensis* (400 mg/kg); NHTG, *n*-hexane extract of *T. gharuensis* (400 mg/kg); piroxicam (10 mg/kg). Values were denoted as mean ± SEM.

## Data Availability

Data used to support the findings of this study are available on request.
